# Dyadic coping after cancer diagnosis – a longitudinal cluster analysis

**DOI:** 10.2340/1651-226X.2025.42561

**Published:** 2025-03-18

**Authors:** Anne-Kathrin Köditz, Anja Mehnert-Theuerkauf, Ute Goerling, Tanja Zimmermann, Beate Hornemann, Franziska Springer, Michael Friedrich, Jochen Ernst

**Affiliations:** a Department of Medical Psychology and Medical Sociology, University Medical Center Leipzig, Leipzig, Germany, Comprehensive Cancer Center Central Germany (CCCG); b Charité – Universitätsmedizin Berlin, Corporate Member of Freie Universität Berlin, Humboldt-Universität zu Berlin, Berlin Institute of Health, Charité Comprehensive Cancer Center, Berlin, Germany; c Department of Psychosomatic Medicine and Psychotherapy, Hannover Medical School, Hannover, Germany; d Comprehensive Cancer Center, University Clinic Centre Dresden, Germany

**Keywords:** Trajectory, partnership, survivorship, couple-related, psycho-oncological

## Abstract

**Background and purpose:**

Dyadic coping (DC) considers the perception of both the individual and their partner’s coping behavior and influences various health outcomes. Given the paucity of research investigating the course of DC after a cancer diagnosis, we explored longitudinal data to find statistically distinct trajectories of DC and to characterize and predict those based on medical, psychological and sociodemographic characteristics.

**Materials and methods:**

In this prospective, multicenter study, we assessed patients with primary solid tumors at four measurement points using validated self-report questionnaires: first within 8 weeks of diagnosis, then at 6-month intervals. We measured DC using the Dyadic Coping Inventory (DCI). Clusters were identified via a feature-based clustering approach, characterized with t-tests and chi-squared tests and predicted with multinomial logistic regression.

**Results and interpretation:**

We analyzed data from 418 patients in a partnership (mean age 61 years, 55.3% men, 84.8% married). Most prevalent cancers were prostate cancer (25.6%), skin cancer (17.5%) and breast cancer (16.3%). One cluster (33.5%) reported a stable high trajectory of coping behavior, indicating good coping behavior. It had the following characteristics: male (62.9%), regularly employed (57.9%), prostate cancer (34.3%) and childless (27.1%). The remaining sample contained a cluster with increasing coping behavior (34.7%) and another with decreasing coping behavior (31.8%). Lack of regular employment, having children and generalized anxiety are significantly associated with worsening coping behavior.

This study is one of the first to examine DC trajectories in a large sample of cancer patients in the early phase after diagnosis. It is essential to understand markers such as psychological stress or family and work-related issues to optimize clinical and psycho-oncological outcomes and facilitate the support or maintenance of couple-related disease management in the long term.

## Introduction

Cancer has increasingly been discussed as a ‘we-disease’, affecting both partners equally [1]. On the one hand, there is a protective effect of a partnership: The prevalence of mental disorders is demonstrably lower among cancer patients who live in a partnership, compared to those who do not [2]. Nevertheless, cancer also presents a significant challenge for couples in terms of adapting to the various illness and treatment related changes. Couples experience positive aspects, such as enhanced cohesion and strength, as well as negative aspects, particularly relating to distressing subjects such as fear, death and dying. The Dyadic Coping Inventory (DCI) is a useful construct to describe this complex process of coping with stress in dyads. It takes account of the fact that the coping strategies of both partners influence each other and records how the couple deals with stress, both at the behavioral level (what both partners do) and at the cognitive level (what both perceive).

This instrument enables us to differentiate the coping behavior into positive (e.g. being close, giving advice, take over tasks) or negative forms (e.g. being superficial, ambivalent, hostile), and into positive coping as a joint effort (to calm down, to find a solution). Furthermore, stress communication (regarded as a trigger for a coping response) and a coping evaluation (satisfaction with and effectiveness of the couple’s coping) can be assessed using the DCI [3].

The link between DC and cancer adjustment is well-established. Previous research has shown that DC is an important factor influencing relationship satisfaction, quality of life as well as the physical or psychological burden of cancer patients [4–7]. There is a paucity of research examining the trajectory of DC following a cancer diagnosis utilizing longitudinal data. Existing studies focus on the relationship between DC and a range of outcomes, including dyadic adjustment, relationship quality or depressive symptoms [8, 9] or consider only one subscale of DC outside the context of cancer [10, 11]. The studies related to cancer report the time since diagnosis, but the way DC develops over time in oncological patients remains unknown. However, an understanding of the trajectory after a diagnosis could facilitate the provision of more personalized psycho-oncological care for cancer survivors and their partners.

It can be assumed that DC is not constant, but follows a course with lows and highs, as it is a response to everyday problems as well as life-changing events. For example, Stadelmann et al. [11] observed increasing negative DC and decreasing positive DC over time for couples in parenthood. A cancer diagnosis represents an existential threat with a chronic character. If these new challenges are matched by the existing coping behavior, a more stable course is to be expected. If they exceed the coping behavior, the DC may improve or deteriorate over time, depending, for example on personal burdens (everyday, psychological, medical etc.).

Therefore, we aim to investigate the trajectory of the coping behavior in patients with solid tumors up to 2 years after cancer diagnosis focusing on the patients’ perspective on the coping behavior. We address the following questions:

Which clusters of trajectories of coping behavior are statistically evident (trajectory clustering)?

What are the distinguishing characteristics of the clusters in terms of medical, psychological and socio-demographic factors and which characteristics predict cluster membership (characteristics of clusters and prediction of cluster membership)?

## Materials and methods

### Data collection

We collected data as a part of the prospective multi-center study ‘Prevalence of mental disorders, psychosocial distress and need for psychosocial support in cancer patients and their relatives stratified by biopsychosocial factors’ (‘LUPE’-study; 2020–2024; study centers in Leipzig, Berlin and Hannover as well as cooperating cancer centers in Braunschweig, Dresden, Göttingen) across four measurement points: first within 8 weeks after diagnosis (follow-up at 6, 12 and 18 months [12, 13]).

Adult patients (≥ 18 years) with the initial diagnosis of a malignant solid tumor were eligible to participate in the study, received a set of validated questionnaires and were interviewed using the Structured Clinical Interview for DSM-5 Disorders (SCID-5-CV).

### Measures

#### Sociodemographic and medical data

We collected socio-demographic characteristics, including age, gender, employment status, information about the partnership (duration of partnership, living in the same household, having children) and information on socio-economic status (SES). SES was determined by taking into account income, education and occupation, according to Winkler and Stolzenberg [14]. Medical data, including tumor entity, Union for International Cancer Control (UICC) disease stage and time since diagnosis, were recorded in questionnaires and extracted from medical records. Based on the SCID-5-CV, we defined the variable ‘SCID diagnoses’, which includes the values 0 = no SCID-5-diagnosis and 1 = one or more SCID-5-diagnoses.

#### Dyadic Coping Inventory

We assessed DC using the DCI [15]. This Instrument is validated in the general population [3], in a sample of university students [16] and is also used in cancer patients [17, 18]. It consists of 10 subscales, of which eight capture the coping behavior (response) and the stress-communication (trigger of the response) from two different perspectives: on the one hand as one’s own coping behavior and on the other hand as the coping behavior of the partner from one’s own perspective. The coping behavior is differentiated into positive DC and negative DC. The positive DC comprises the supportive DC and the delegated DC. Thus, coping behavior is covered twice by three subscales: supportive (five items), delegated (two items) and negative DC (four items), each considered from the two perspectives. Furthermore, there are two subscales for stress communication, each with four items and considered from the two perspectives. Then there is one subscale which assesses positive coping as a joint effort (common DC, five items) and one subscale which assesses the quality of the coping behavior (evaluated DC, two items). The response options from all items range from 1 (very rarely) to 5 (very often) and we form subscales using the mean value of the items. Higher values represent a more intense coping [3, 19].

In this study, we focused on the coping *behavior*. Therefore, we report on the subscales of supportive DC, delegated DC and negative DC for the own behavior and the partner’s behavior respectively, as well as the common DC. The values for Cronbach’s alpha at t1 in our study were as follows: supportive DC 0.83 (own behavior)/0.86 (partner’s behavior), delegated DC 0.81/0.79, negative DC 0.71/0.68, common DC 0.86. To identify trajectories of the patient’s coping behavior, we combined the first three mentioned subscales for the patient’s own behaviors using the mean values of the subscales into a total score for the patient’s own coping behavior, with the negative DC inverted (Cronbach’s alpha in our study: 0.68).

The subscales for stress communication and evaluated DC are not part of this patient-centered view of coping behavior.

#### Other instruments

We examined the following psychosocial characteristics through validated questionnaires. To assess psychological distress we used a one-item screening tool for cancer patients (Distress-thermometer, DT, range: 0 = not burdened at all to 10 = extremely burdened; [20]). Fear of progression was assessed using the Fear of Progression Questionnaire-Short Form (FoP-Q-SF, range: 1 = never to 5 = very often; [21], Cronbach’s alpha in our study at t1: 0.88), symptoms of depression with the Patients Health Questionnaire (PHQ-9, range: 0 = not at all to 3 = almost every day; [22], Cronbach’s alpha in our study at t1: 0.86). Anxiety was measured using the questionnaire ‘General Anxiety Disorder’ (GAD-7, range: 0 = not at all to 3 = almost every day; [23], Cronbach’s alpha in our study at t1: 0.88), and relationship satisfaction with the Quality of Marriage Index (QMI, 1 = strong rejection to 7 = strong agreement; [24, 25], Cronbach´s alpha in our study at t1: 0.97).

### Statistical analyses

#### Trajectory clustering

To identify groups with different trajectories of DC, we used a feature-based method of longitudinal clustering [26]. In order to identify the best cluster solution, we based our decision on the silhouette coefficient [27]. It ranges from –1 to +1. Higher values indicate that the corresponding object is closer to its own cluster, while negative values indicate that it is closer to an object of another cluster. Average values from –1 to 0.2 indicate a poor quality, values from 0.2 to 0.5 a fair quality and values from 0.5 to 1 a good quality of the solution [28].

#### Cluster differences and predictors of cluster membership

Differences between the trajectory clusters were evaluated by chi-squared tests (for categorical characteristics) and t-tests (for continuous characteristics) for each pair of groups. To control for type-I-error-inflation due to multiple testing, we present post-hoc corrected p-values using the Games–Howell procedure for the *t*-tests [29]. To judge the magnitudes of effect size we present Cohen’s omega and Hedges’ g [30, 31]. Cohen’s omega is considered a correlation-equivalent effect size and is often used when analyzing cross-tabulations (categorical characteristics). Effects of around 0.1 can be classified as weak, around 0.3 as medium and around 0.5 as large. Hedges’ g is a bias-corrected version of Cohen’s d for mean comparisons (continuous characteristics), which corrects for an overestimation of the effect size as the group size decreases. Effects around 0.2 can be classified as weak, around 0.5 as medium and around 0.8 as strong.

We performed a stepwise multinomial logistic regression to predict the trajectory cluster membership. The following characteristics were included: socio-demographic (age, sex, SES, employment status, residential area, marital status, duration of partnership, children), medical (diagnostic group, cancer stage [UICC], SCID-diagnosis), coping-related (patient’s perspective on the partner’s positive, negative and delegated DC as well as the patient’s perspective on common DC) and psychological (distress, anxiety, depression, fear of progression and relationship satisfaction).

In order to establish a reference category, the characteristic with the highest frequency was selected from the set of characteristics that were summarized. Initially, we started with the socio-demographic, medical and psychological characteristics, then predictors without a significant contribution were excluded stepwise.

For statistical analyses we used r (version 4.4.1, [32]) with the packages *cluster* (version 2.16, [33]) to determine the cluster membership and *ggplot2* (version 3.5.1, [34]) graphical representations, and a package with implementations of Vector Generalized Linear and Additive Models, (VGAM, version 1.1-12, [35] for performing the multinomial logistic regression.

## Results

### Sample characteristics

In total 1,702 eligible patients were contacted, of whom *n* = 1,150 participated in the study. A total of 842 patients of this sample are in a partnership and 418 patients completed the DCI at all measurement time-points (t1–t4) and were included in our analysis.

Goerling et al. [13] analyzed the data of the patients who were excluded from or who refused to participate. Compared to study participants, excluded patients were older, more likely to be male and differed in cancer entities. Characteristics of patients who refused to participate (most frequent reasons: not interested, too burdensome, organizational barriers) are also a higher age, a worse performance status and a lower SES.

Sociodemographic and medical characteristics of our sample are presented in Table 1.

**Table 1:** Sample characteristics (*N* = 418).

Characteristic	*N* *	(%)
**Age**		
Mean (SD; range)	60.5 (12.4; 25–88)	
**Gender**		
Female	187	(44.7)
Male	231	(55.3)
**Socioeconomic status (SES)**		
High	191	(45.8)
Middle	191	(45.8)
Low	35	(8.4)
**Employment status**		
Employed (full- or part-time)	210	(50.2)
Retired	175	(41.9)
Unemployed	7	(1.7)
Other	8	(1.9)
**Residential area**		
Urban (> 20,000 inhabitants)	223	(54.9)
Rural (< 20,000 inhabitants)	181	(44.8)
**Marital status**		
Married	341	(84.8)
Not married	61	(15.2)
**Duration of partnership (years)**		
Mean (SD; range)	31.74 (16.3; 0–32)	
**Living in same household**		
Yes	378	(95.0)
No	20	(5.0)
**Children**		
Yes	329	(82.3)
No	71	(17.8)
**Diagnostic group (ICD-10)**		
Prostate (C61)	107	(25.6)
Skin (C43, C44)	73	(17.5)
Breast (C50)	68	(16.3)
Gynecological (C51–C54, C56, C57)	49	(11.7)
Gastrointestinal (C15–18, C20–C22, C24, C25)	39	(9.3)
Head and neck (C00–C03, C6, C7, C09, C10, C30–C32)	26	(6.2)
Urinary tract (C64–C67)	29	(6.9)
Other (C34, C41, C48, C49, C60, C62, C71,C74, C80, C97)	27	(6.5)
**Cancer stage (UICC)**		
I / II	307	(77.5)
II/III	89	(22.5)
**SCID diagnoses (any mental disorder)**		
Yes	71	(18.0)
No	323	(82.0)
**Additional psychological characteristics at t1 (Mean (SD; range))**		
Distress	3.8 (2.6; 0–10)	
Anxiety	4.1 (4.0; 0–21)	
Depression	5.6 (4.7; 0–27)	
Fear of progression	29.0 (9.3; 12–58)	
Relationship satisfaction	40.8 (6.1; 6–45)	

* Difference to 418 = missing values.

#### Trajectory clustering

**Figure 1:**
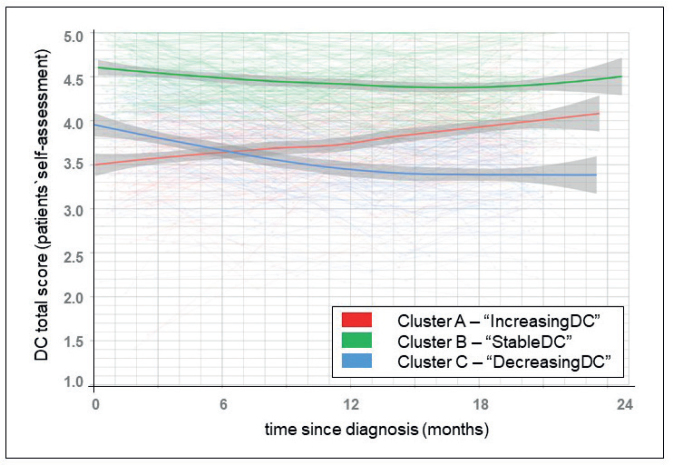
Trajectory of dyadic coping (DC) for patients over 2 years after cancer diagnosis.

The average silhouette values ranged from 0.30 (eight clusters) to 0.34 (three clusters), indicating a three-cluster solution as the best approach with cluster sizes of *n* (%) in cluster A = 145 (34.7%), B = 140 (33.5%) and C = 133 (31.8%). The solution can be classified as fair and is a statistically acceptable solution to represent trajectory clusters in our data. The trajectory of the DC within each cluster is presented in Figure 1. While cluster B follows a stable course, the two remaining clusters show a variable trajectory, which always yields lower DC scores over the observation period than those in the stable cluster.

Cluster A starts off with a slightly lower DC score than cluster C. As the trend in cluster A tends to increase, but decreases in cluster C, the DC scores in months 5 to 8 appear to be at a similar level, so that towards the end of the first year after diagnosis, the DC score in cluster A is higher than the DC score in cluster C (in the following: cluster A = ‘IncreasingDC’, cluster B = ‘StableDC’, cluster C = ‘DecreasingDC’).

#### Cluster differences and prediction of cluster membership

Table 2 shows the comparisons of the clusters with the categorical characteristics at baseline. A weak association with cluster affiliation was identified for the gender of the patients: Chi² (df) *p* = 7.4 (2), 0.024, Cohen’s omega = 0.13. The largest proportion of male respondents was found in cluster ‘StableDC’, with 62.9%. Furthermore, the cluster ‘StableDC’ comprised the smallest proportion of patients being retired, unemployed or with another status of employment (42.1%) and the highest proportion of prostate cancer patients (34.3%) and childless patients (27.1%). The cluster ‘IncreasingDC’ contained the largest proportion of gynecological tumors (15.2%). No significant association with cluster membership could be shown for SES, marital status, SCID-diagnosis or cancer stage.

**Table 2:** Associations between categorical characteristics at t1 and trajectory cluster membership.

Characteristic [Reference]	*n* (%)			Chi² (df), *p*	Cohen’s omega
	‘IncreasingDC’ 145 (34.7)	‘StableDC’ 140 (33.5)	‘DecreasingDC’ 133 (31.8)		
**Gender:** female [male]	77 (53.1)	52 (37.1)	58 (43.6)	**7.4 (2), 0.024**	0.133
**SES: high** [low or medium]	59 (40.7)	77 (55.0)	55 (41.4)	7.8 (4), 0.098	0.137
**Employment status:** retired, unemployed, other [employed]	73 (50.3)	59 (42.1)	76 (57.1)	**6.2 (2), 0.046**	0.121
**Residential area:** rural [urban]	84 (57.9)	80 (57.1)	73 (54.9)	0.3 (2), 0.870	0.026
**Marital status:** not married [married]	28 (19.3)	30 (21.4)	19 (14.3)	2.4 (2), 0.296	0.076
**Living in same household:** yes [no]	131 (95.6)	123 (93.9)	124 (95.4)	0.5 (2), 0.784	0.035
**Children:** no [yes]	32 (22.1)	38 (27.1)	19 (14.3)	**6.8 (2), 0.033**	0.128
**Cancer diagnostic group**					
**Prostate** [else]	27 (18.6)	48 (34.3)	32 (24.1)	**9.4 (2), 0.009**	0.150
**Skin** [else]	24 (16.6)	27 (19.3)	22 (16.5)	0.5 (2), 0.785	0.034
**Breast** [else]	26 (17.9)	23 (16.4)	19 (14.3)	0.7 (2), 0.712	0.040
**Gynecological** [else]	22 (15.2)	10 (7.1)	17 (12.8)	4.6 (2), 0.098	0.105
**Gastrointestinal** [else]	16 (11.0)	10 (7.1)	13 (9.8)	1.3 (2), 0.517	0.056
**Other** [else]	30 (20.7)	22 (15.7)	30 (22.6)	2.2 (2), 0.335	0.072
**SCID-diagnosis:** yes [no]	25 (17.2)	21 (15.0)	25 (18.8)	0.7 (2), 0.702	0.041
**UICC cancer stage:** III-IV[I-II]	30 (20.7)	24 (17.1)	35 (26.3)	3.5 (2), 0.176	0.091

DC: dyadic coping; SES: socioeconomic status; SCID: Structured Clinical Interview; UICC: Union for International Cancer Control; Chi²: chi-squared statistic; df: degrees of freedom; p: type-I-error probability; Cohen’s omega: effect size. Significant results (p-value < 0.05) are marked in bold.

Table 3 shows the comparisons of the clusters with regard to the continuous characteristics at baseline. Except for age and duration of partnership there was at least one significant difference between two clusters for each characteristic. The largest effect among the weak to moderate effects for the for the non coping-related characteristics was observed for the relationship satisfaction: Cluster ‘StableDC’ with a value of M (SD) = 43.1 (4.7) differs from cluster ‘DecreasingDC’ with a value of 39.2 (6.9) by Hedges’ g = –0.67 standard deviations. The *t*-test was significant: t (df), *p* = 5.5 (230.5), <0.001.

**Table 3:** Differences between trajectory clusters.

Characteristics [t1]	‘IncreasingDC’ 145 (34.7%)	‘StableDC’ 140 (33.5%)	‘DecreasingDC’ 133 (31.8%)	△ ‘StableDC’/ ‘IncreasingDC’	△ ‘DecreasingDC’/ ‘IncreasingDC’	△ ‘DecreasingDC’/ ‘StableDC’	△ ‘StableDC’/ ‘IncreasingDC’	△ ‘DecreasingDC’/ ‘IncreasingDC’	△ ‘DecreasingDC’/ ‘StableDC’
	m (sd)			t (df), p			Hedges’ g		
**Age**	60.2 (12.5)	59.2 (12.8)	62.2 (11.8)	0.7 (282.0), 0.771	1.4 (275.7), 0.343	2.1 (270.8), 0.100	-0.08	0.17	0.25
**Duration of partnership** [years]	32.2 (15.6)	29.3 (16.0)	33.7 (15.9)	1.5 (282.0), 0.274	0.8 (273.1), 0.714	2.3 (270.5), 0.063	-0.18	0.09	0.27
**Distress**	3.5 (2.6)	3.5 (2.5)	4.4 (2.7)	0.1 (282.0), 0.989	**2.7 (268.4), 0.022**	**2.8 (265.2), 0.015**	-0.02	0.32	0.34
**Anxiety**	4.2 (3.9)	3.0 (3.4)	5.1 (4.4)	**2.7 (280.9), 0.019**	1.8 (262.1), 0.178	**4.3 (247.3), <0.001**	-0.32	0.21	0.53
**Depression**	6.1 (5.0)	4.3 (3.9)	6.3 (4.8)	**3.4 (270.8), 0.002**	0.4 (274.0), 0.910	**3.8 (251.4), <0.001**	-0.40	0.05	0.47
**Fear of progression**	29.3 (9.0)	27.1 (9.0)	30.7 (9.6)	2.1 (282.5), 0.097	1.2 (266.1), 0.427	**3.2 (264.4), 0.005**	-0.25	0.15	0.39
**Relationship satisfaction**	39.9 (5.9)	43.1 (4.7)	39.2 (6.9)	**5.0 (272.9), <0.001**	1.0 (260.0), 0.572	**5.5 (230.5), <0.001**	0.59	-0.12	-0.67
**Patients supportive DC** [patients perspective]	3.7 (0.7)	4.5 (0.4)	3.8 (0.7)	**12.6 (245.4), <0.001**	1.3 (275.6), 0.423	**11.1 (228.8), <0.001**	1.47	0.15	-1.36
**Patients delegated DC** [patients perspective]	145, 4.0 (0.8)	140, 4.6 (0.5)	133, 4.0 (0.9)	**15.6 (245.4), <0.001**	**6.8 (274.0), <0.001**	**8.8 (245.5), <0.001**	0.88	-0.03	-0.85
**Patients negative DC** [patients perspective]	145, 2.0 (0.6)	140, 1.3 (0.4)	133, 1.9 (0.8)	**11.8 (251.8), <0.001**	2.0 (247.0), 0.117	**7.1 (194.3), <0.001**	-1.38	-0.24	0.87
**Partners supportive DC** [patients perspective]	145, 1.7 (0.6)	140, 1.3 (0.4)	132, 1.8 (0.8)	**7.5 (253.5), <0.001**	0.2 (264.7), 0.968	**6.9 (213.5), <0.001**	-0.75	0.16	0.79
**Partners delegated DC** [patients perspective]	145, 3.0 (1.0)	140, 4.4 (0.6)	133, 3.7 (0.8)	**4.6 (257.2), <0.001**	0.7 (267.0), 0.786	**4.9 (220.4), <0.001**	1.83	0.81	-1.07
**Partners negative DC** [patients perspective]	145, 4.2 (0.9)	140, 4.6 (0.6)	133, 4.1 (1.0)	**6.4 (264.2), <0.001**	1.3 (239.5), 0.398	**6.4 (199.0), <0.001**	0.54	-0.08	-0.60
**Common DC** [patients perspective]	145, 3.3 (0.8)	140, 4.1 (0.6)	133, 3.3 (0.8)	**9.4 (270.7), <0.001**	0.1 (273.7), 0.992	**9.3 (248.4), <0.001**	1.11	-0.01	-1.13

DC: dyadic coping; n: group size; m: mean; sd: standard deviation; t: t-value; df: degrees of freedom; p: type-I-error probability; Hedges’ g standardized mean difference. Significant results (p-value < 0.05) are marked in bold.

With regard to the baseline DC-scores the stable cluster differs from the unstable clusters (increasing, decreasing) in all subscales (supportive, delegated and negative DC for the patient’s own and the patient’s perception of the partner’s DC) with effect sizes (Hedges’ g) from 0.54 (perception of partner’s negative DC, in comparison ‘IncreasingDC’ vs. ‘StableDC’) to 1.83 (perception of partner’s delegated DC, in comparison ‘IncreasingDC’ vs. ‘StableDC’). No differences were observed between the cluster with the increasing trajectory and the one with the decreasing trajectory except for the patient’s own delegated DC, but with a negligible effect (*g* = 0.03 in comparison ‘IncreasingDC’ vs. ‘DecreasingDC’).

The analysis of the predictors (baseline) of cluster membership is presented in Table 4. The decreasing cluster was chosen to be the reference group against which the other trajectory clusters were compared. With lower scores in the negative DC and higher scores in the common and delegated DC at baseline, the chance of membership of the ‘StableDC’ cluster increases. Patients who are unemployed, have children or report higher levels of generalized anxiety are more likely to belong to cluster ‘DecreasingDC’. With increasing everyday stress (distress), the chance of being in the increasing cluster decreases significantly by a factor of 0.88 per scale point. The explained variance of the model is Nagelkerke’s pseudo *R*^2^ = 0.339.

**Table 4:** Multinomial logistic regression for predicting trajectory cluster membership.

	‘IncreasingDC’ versus ‘DecreasingDC’		‘StableDC’ versus ‘DecreasingDC’	
	B (SE), p-value	OR (95%CI)	B (SE), p-value	OR (95%CI)
**Intercept**	1.19 (0.97), 0.219		–**4.58 (1.37), <0.001**	
**Partners’ negative DC** [t1, patients perspective]	–0.12 (0.20), 0.551	0.89 (0.60–1.31)	–**0.89 (0.30), 0.003**	**0.41 (0.23–0.73)**
**Partners’ delegated DC** [t1, patients perspective]	0.13 (0.15), 0.398	1.14 (0.84–1.54)	**0.49 (0.21), 0.020**	**1.63 (1.08–2.47)**
**Common DC** [t1, patients perspective]	–0.19 (0.18), 0.284	0.82 (0.58–1.17)	**1.21 (0.22), <0.001**	**3.34 (2.15–5.19)**
**Employment status: unemployed** [reference: full-/part-time]	–0.46 (0.26), 0.079	0.63 (0.37–1.06)	–**0.70 (0.30), 0.019**	**0.50 (0.28–0.89)**
**Children: no** [reference: yes]	0.56 (0.33), 0.091	1.75 (0.91–3.34)	**0.73 (0.36), 0.044**	**2.07 (1.02–4.18)**
**Distress**	–**0.13 (0.06), 0.027**	**0.88 (0.79–0.99)**	–0.02 (0.07), 0.796	0.98 (0.87–1.12)
**Anxiety**	–0.03 (0.04), 0.479	0.97 (0.90–1.05)	–**0.13 (0.05), 0.006**	**0.88 (0.80–0.96)**

Notes: Chi² (df) = 148.1 (14), *p* < 0.001, AIC = 790.4, *R*² = 0.301 (Cox & Snell), 0.339 (Nagelkerke), 0.163 (Mc Fadden).

DC: dyadic coping; B: regression coefficient; SE: standard error; p: type-I-error probability; OR: odds ratio; CI: confidence interval. Significant results (p-value < 0.05) are marked in bold.

## Discussion

This study is one of the first to investigate the trajectory of DC in a large sample with solid tumors over 2 years. We were able to identify three coping trajectories (stable, increasing, decreasing). The only comparative study on coping trajectories [11] identified stable high and moderately decreasing courses after parenthood. This is also an incisive life event, which can be attributed an existential character, but lacks the life-threatening element that a cancer diagnosis possesses.

Overall, the patients in the sample showed relatively high values for the DC score, which are similar to those reported by other studies with cancer patients [36]. Patients with a higher DC value at the first measurement time-point were more likely to show a stable high DC trajectory. The unstable clusters in our study start at a similar value – which is lower than for the stable high trajectory – but differ at the end of the observation period. This indicates that patients who initially demonstrate good DC may often maintain this coping behavior over time. Conversely, for patients with a lower score, the trajectory of DC can be influenced by personal burdens, resulting in either an improvement or deterioration over time.

The results of our analysis show that the DC clusters differ on several socio-demographic, medical and psychological variables. The cluster ‘StableDC’ included patients with potentially less burden: fewer material concerns due to employment, a lack of child care duties and of worries about the children’s future. Furthermore, the relationship satisfaction was highest in the cluster with the stable high DC trajectory, consistent with previous research, which identified a positive correlation between relationship satisfaction and DC [5]. Additional distress in turn seems to make it more difficult to improve coping behavior: these patients were more likely to be in the decreasing DC cluster, indicating an association between higher distress and lower DC scores [37].

Burdens like unemployment or higher levels of generalized anxiety as well as having children can also lead to deteriorating DC after a diagnosis of cancer. Knowledge of these features, which characterize stable or increasing DC trajectories, provides clinicians with clues to potentially relevant issues that should be considered when taking a medical history. This link between medical, sociodemographic and psychological characteristics and a change in the course of coping could enable targeted couple support for cancer survivors and their partners and strengthen their partnership as a resource for mental health [2].

## Limitations

The results of our study could be biased by the lower proportion of participants with low SES (8.4%). The lower level of education and poorer access to information about their illness and support services could also have an influence on DC of the couple. Thus, people with low SES could be more burdened and decreasing trajectories of DC could be underrepresented in our sample. The fit of our cluster solution was ‘fair’ and statistically acceptable. This result is based on the fact that the DC follows an individually erratic course. As a result, homogeneity within the clusters is limited and the clusters cannot be clearly distinguished from one another. This can mask mean differences between the clusters and makes it more difficult to predict cluster membership. Further research could validate our cluster solution using a comparable sample of cancer patients and could identify other characteristics which are associated with cluster membership. Furthermore, we have examined a patient-centered view of DC here. In further work, the focus could be placed on the perspective of the partners. As in other studies, there were more men among the non-participants, but also older patients who did not take part in the study. This limits the generalizability of our results.

## Conclusion

Two thirds of patients reported a good process of DC (stable, increasing). For about one third, coping deteriorates, indicating a reduction in coping behavior, an increase in stress or anxiety or the need to adapt to the cancer due to symptoms, treatment settings or side effects. It is essential to understand markers such as psychological stress or family and work-related issues to optimize clinical and psycho-oncological outcomes and facilitate the support or maintenance of couple-related disease management in the long term.

## Data Availability

The data that support the findings of this study are available from the corresponding author, upon reasonable request.
